# Epidemiology, Pathogenesis, Clinical Manifestations, and Management Strategies of Tuberculous Meningitis

**DOI:** 10.26502/aimr.0195

**Published:** 2025-02-10

**Authors:** Nicholas Oo, Devendra K. Agrawal

**Affiliations:** 1 Department of Translational Research, College of Osteopathic Medicine of the Pacific, Western University of Health Sciences, Pomona, California 91766 USA

**Keywords:** Drug resistance, Granuloma, Host-directed therapy, Immune modulation, Mycobacterium, Neurotoxicity, Oxidative stress, Public health, Tuberculosis, Tuberculous meningitis

## Abstract

Tuberculous meningitis (TBM), the most severe manifestation of extrapulmonary tuberculosis, poses significant global health challenges due to its high mortality rates and complex pathophysiology. This review synthesizes recent findings on TBM, covering epidemiology, pathogenesis, clinical manifestations, diagnostics, and management strategies. TBM disproportionately affects immunocompromised populations, including individuals with HIV, with the highest mortality observed in low-resource settings. Pathogenesis involves Mycobacterium tuberculosis breaching the blood-brain barrier, eliciting a granulomatous inflammatory response that contributes to neurotoxicity. Advances in diagnostics, such as next-generation sequencing and novel imaging techniques, have improved early detection and treatment guidance. Management strategies emphasize multidrug regimens, adjunctive corticosteroids, and emerging therapies like intrathecal administration and nanoparticle-based drug delivery. Host-directed therapies targeting immune modulation and oxidative stress show promise in improving outcomes, particularly for drug-resistant TBM. Despite advancements, diagnostic delays, treatment resistance, and high rates of neurological effects underscore the need for further research. Preventive strategies focusing on early diagnosis, modifiable risk factor management, and public health interventions are critical to reducing global burden of TBM. This review highlights the importance of integrating innovative diagnostics, tailored treatments, and preventive measures to address the challenges of TBM and improve patient outcomes.

## Introduction

Tuberculous meningitis (TBM) is the most severe form of extrapulmonary tuberculosis (TB), characterized by infection and inflammation of the meninges caused by *Mycobacterium tuberculosis* (*M. Tuberculosis)* [1]. The disease represents a substantial health challenge due to its complex pathophysiology and high mortality rates, even in cases where timely treatment is initiated [2]. TBM disproportionately affects immunocompromised populations, particularly those living with HIV, further compounding the global burden of tuberculosis [3].

Recent research has highlighted the interplay of genetic predispositions and immune responses that contribute to TBM susceptibility and progression [4]. Moreover, the nonspecific clinical presentation of TBM and diagnostic difficulties, such as the need for advanced tools and delayed detection, remain significant barriers to timely management. This review synthesizes the latest findings on TBM’s epidemiology, pathogenesis, clinical manifestations, diagnostic approaches, management strategies, prognosis, prevention, and research gaps to provide a comprehensive overview of this critical condition.

### Epidemiology of Tuberculous Meningitis

Tuberculous meningitis accounts for 1% of all tuberculosis cases but contributes disproportionately to TB-related deaths [5]. A recent meta-analysis indicates a global TBM mortality rate of approximately 25%, with mortality as high as 70% in sub-Saharan Africa, highlighting significant geographic disparities in disease outcomes [5]. These differences are often linked to variations in healthcare access, diagnostic capacity, and treatment availability [5].

Certain populations face an elevated risk of developing TBM [3]. Among people living with HIV, TBM prevalence is notably high, driven by compromised immune responses that hinder the containment of *M. tuberculosis* [3]. Advanced age has also been identified as a significant risk factor, with older individuals experiencing worse outcomes due to delayed diagnosis and weaker immune defenses [1]. These challenges are further compounded by conditions like malnutrition, which not only increases susceptibility to severe forms of TB but is also more prevalent in regions experiencing poverty and limited healthcare resources [6].

In addition, socioeconomic factors deeply influence TBM outcomes. Malnutrition serves as a critical example, linking poor nutritional status to weakened immune defenses and higher risks of progression to severe disease. These effects are amplified in low-resource settings where healthcare access is restricted, diagnostic delays are common, and interventions are inadequate [3,6]. Addressing these intertwined challenges requires comprehensive public health strategies aimed at improving nutrition, healthcare infrastructure, and early diagnosis.

### Pathogenesis of Tuberculous Meningitis

**Figure 1:**
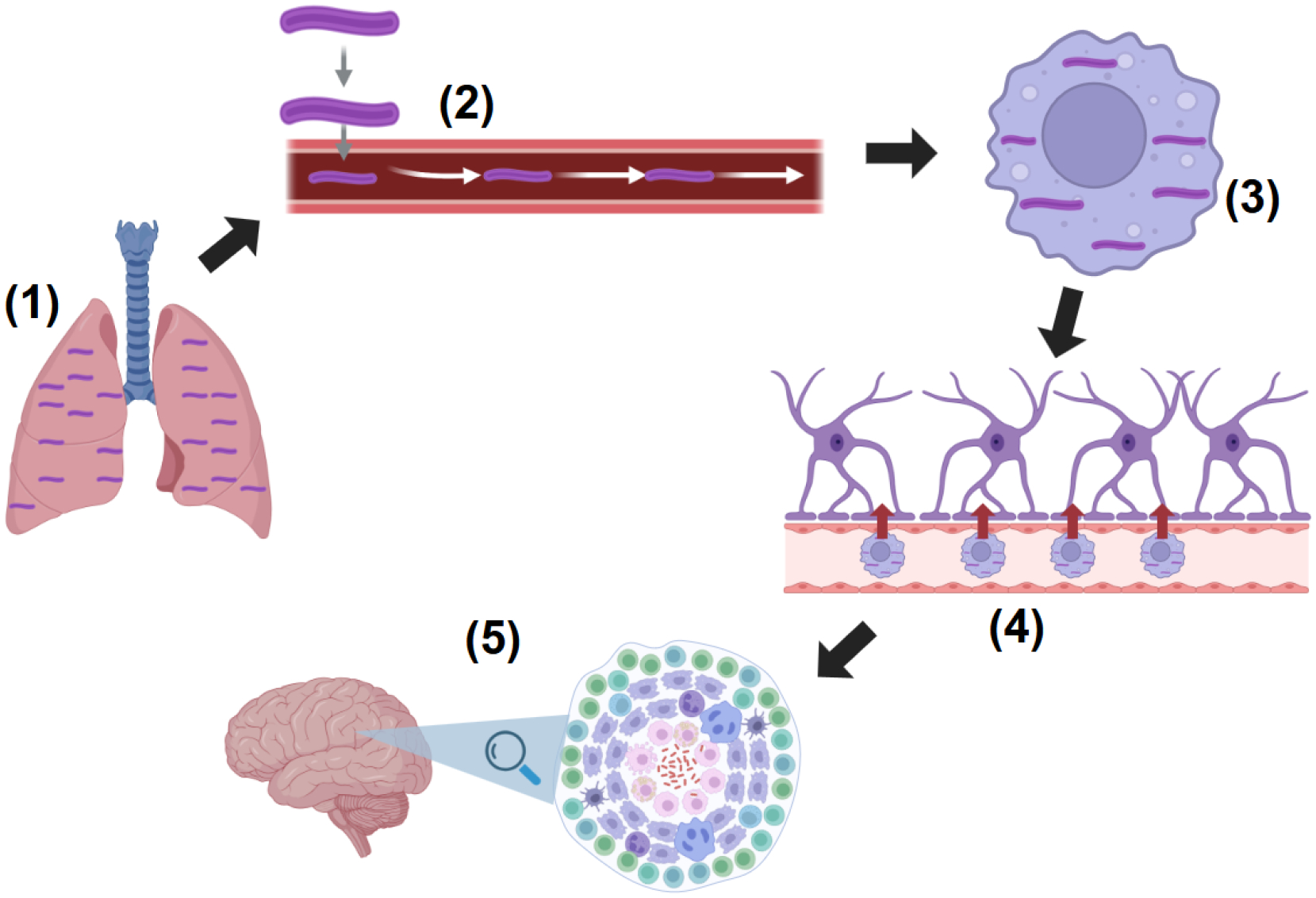
Overview of the pathogenesis of TBM. (1) Primary infection of lungs by M. tuberculosis. (2) M. tuberculosis disseminates through bloodstream. (3) Phagocytosis of M. tuberculosis by macrophages. (4) Macrophages cross BBB with M. tuberculosis to CNS and interact with microglia, triggering immune response and inflammation. (5) M. tuberculosis creates granulomas, known as Rich foci, which can rupture into subarachnoid space, further spreading infection. Created with BioRender.com

The pathogenesis of TBM involves a complex interplay between host immune responses and the virulence mechanisms of *M. tuberculosis* (Figure 1}. After systemic infection, *M. tuberculosis* disseminates through the bloodstream and breaches the blood-brain barrier (BBB) where bacteria infiltrate the CNS via macrophages [7]. Bacterial virulence factors, including those that facilitate actin rearrangement and adhesion, aid in BBB traversal. Once inside the CNS, *M. tuberculosis* establishes a leptomeningeal or cortical granuloma, known as a “Rich focus,” which can rupture into the subarachnoid space, spreading infection and triggering intense inflammation [8]. This immune response, characterized by neutrophil-driven inflammation and cytokine release, balances bacterial control and tissue damage [2]

Macrophages and microglia play central roles in TBM [7]. Microglia are resident immune cells of the CNS that respond to M. tuberculosis invasion by releasing pro-inflammatory cytokines like TNF-α and IL-1β, which control bacterial replication but can also lead to neuroinflammation and tissue injury [7]. Prolonged activation of microglia may result in neurotoxicity and cognitive impairments [7]. Granuloma formation is a hallmark of TBM that arises from immune cell aggregation around M. tuberculosis and often leads to vascular damage in advanced stages [7] (Figure 1).

Genetic predisposition significantly influences TBM susceptibility [4]. Variations in genes regulating immune responses, such as those coding for surface receptors, transcription factors, and signaling molecules, are linked to an increased risk of disease [4]. Specific mutations in genes such as IFNGR1, IFNGR2, STAT1, IL12RB1, and TYK2 disrupt cytokine signaling and immune cell activation, impairing the host’s ability to mount an effective response [4]. Defects in the IL-12/IFN-γ axis, crucial for macrophage activation and bacterial control, are strongly associated with TBM susceptibility [4].

Oxidative stress is a pivotal element in TBM pathogenesis [9]. M. tuberculosis counters host oxidative defenses through antioxidant enzymes like superoxide dismutase, catalase-peroxidase, and alkyl hydroperoxide reductase, enabling survival in oxidative environments [9]. Additionally, M. tuberculosis promotes ferroptosis by acquiring host iron, depleting GPX4, and inducing lipid peroxidation, which exacerbates neuronal damage and inflammation [9]. Nitric oxide, a key reactive nitrogen species (RNS), reacts with superoxide to form peroxynitrite, a potent antimicrobial that damages M. tuberculosis but also contributes to neuroinflammation and BBB disruption [9].

Recent research highlights the role of indoleamine 2,3-dioxygenase 2 (IDO2) in TBM. IDO2 is abundantly expressed in both granulomatous and non-granulomatous brain tissues, including the parenchyma, inferior olive, and cerebellum [10]. Driven by the aryl hydrocarbon receptor (AHR) pathway, IDO2 metabolizes tryptophan into kynurenine metabolites like quinolinic acid, which are neurotoxic [10]. These metabolites contribute to apoptosis in granulomas and autophagy in non-granulomatous areas, exacerbating neuroinflammation and cellular dysfunction [10]. Elevated IDO2 activity reflects advanced stages of TBM and is associated with broader brain inflammation [10].

MicroRNAs are emerging as crucial regulators of immune and inflammatory pathways in TBM. Dysregulated microRNAs influence the expression of matrix metalloproteinases (MMPs), which play critical roles in BBB disruption and neuroinflammation [11]. For example, downregulation of hsa-miR-495–3p and hsa-miR-132–3p is associated with increased levels of MMP2 and MMP3, respectively, exacerbating extracellular matrix remodeling and leukocyte migration [11]. Conversely, hsa-miR-21–5p is upregulated in TBM, reducing inhibitors of MMPs and amplifying their activity. This dysregulation highlights their potential as biomarkers for TBM diagnosis and progression [11]. Targeting miRNA-MMP interactions offers promising therapeutic strategies to mitigate neuroinflammation and limit TBM complications [11].

### Clinical Manifestations

Tuberculous meningitis is a severe form of extrapulmonary tuberculosis that primarily affects the central nervous system (CNS). Its clinical manifestations are nonspecific, often delaying diagnosis and increasing the risk of severe complications [12,13]. Initial symptoms, such as fever, neck stiffness, headache, and malaise, can progress to neurological signs like altered mental status, cranial nerve palsies, and focal deficits [14,15].

#### Typical Presentations:

Neurological deficits are a hallmark of TBM and can mimic other CNS pathologies [16]. Rapidly progressing neurological symptoms should prompt consideration of TBM [13]. Cranial nerve involvement, particularly affecting the second, third, and sixth nerves, is common in TBM [14]. Increased intracranial pressure is another frequent complication, leading to symptoms such as vomiting and papilledema. Hydrocephalus, a significant contributor to these complications, exerts direct pressure on the optic nerve and reduces its blood flow. This leads to swelling, dysfunction, and eventual atrophy, underscoring the critical need for early intervention [17].

Vascular complications such as vasculitis and stroke are frequently observed in TBM. Vasculitis caused by TBM is a leading cause of stroke, with infarctions commonly affecting regions such as the thalamus and basal ganglia [12]. A rare infarct location in the right basifrontal lobe underscores the diverse cerebrovascular impacts of TBM [12]. Predictors of cerebral infarction include elevated blood pressure and shortened onset-to-treatment intervals [18].

Brain tuberculomas are another prominent feature of TBM. These lesions frequently occur in areas with rich blood supply, such as the cerebral hemispheres, cerebellum, and brainstem, and are often associated with headaches, night sweats, and focal neurological deficits [19]. Conversely, a case of an HIV-positive patient carrying at least 34 tuberculomas asymptomatically before developing fatal TBM demonstrates the potential for extensive yet silent CNS involvement [20]. These lesions can mimic neoplastic or other pathological entities, highlighting the need for histopathological confirmation to establish a definitive diagnosis [21].

Seizures represent another significant clinical manifestation. In TBM patients, seizures occurred in approximately 37.2% of cases, with generalized tonic-clonic seizures being the most frequent, followed by focal seizures and focal to bilateral seizures [22]. Status epilepticus (SE), a severe form of seizure, was observed in 6.9% of TBM patients [15]. Tuberculomas located in cortical areas are particularly likely to trigger seizures due to irritation of grey matter neurons, highlighting the role of lesion location in seizure development [22].

#### Atypical Presentations:

Atypical presentations of TBM, while rare, can complicate diagnosis and management. Psychiatric symptoms, such as depression, hallucinations, and disorientation, can serve as the initial manifestations of TBM [13]. These symptoms may mask the infection and delay diagnosis, especially in patients with pre-existing psychiatric conditions [13]. A rare presentation of TBM involving Broca’s aphasia can occur due to injury in the cortical language center of the brain, specifically the left frontal lobe [23]. This area is responsible for speech production and some motor functions related to language, resulting in significant speech and language impairments and demonstrating the diverse neurological impact of TBM on communication abilities [23].

Isolated spinal TBM, where patients present with radiculopathy, back pain, or paraplegia without classic meningeal signs, highlights the potential for TBM to involve the spinal cord or nerve roots in rare cases [24]. A case of recurrent thoracic spinal intradural arachnoid cysts and intradural abscesses secondary to TBM caused progressive myelopathy and motor deficits, emphasizing the severe neurological complications that can arise [24].

While TBM frequently impacts the visual system, rare presentations like bilateral internuclear ophthalmoplegia (INO) and exotropia have also been documented, highlighting the diverse neuro-ophthalmic effects of TBM [25]. Additionally, bilateral optic neuritis has been reported as the initial presentation of TBM without the classic features like fever and neck stiffness, further complicating timely diagnosis [17].

### Diagnosis of Tuberculous Meningitis

Diagnosing tuberculous meningitis (TBM) is a challenging task due to its nonspecific clinical presentation and the paucibacillary nature of cerebrospinal fluid (CSF) samples. Advances in molecular diagnostics, imaging, and scoring systems have improved diagnostic accuracy, particularly in resource-limited settings.

#### Cerebrospinal Fluid Analysis:

CSF analysis remains one of the best diagnostic tools for TBM diagnosis. Elevated protein levels, low glucose concentration, and lymphocytic pleocytosis are characteristic but nonspecific findings [26]. Adenosine deaminase (ADA) levels, with a sensitivity of 84.5% and specificity of 88.1%, provide a useful biomarker for diagnosis, especially when paired with other tests [27].

#### Molecular Diagnostics:

Molecular tools like GeneXpert MTB/RIF, nanopore sequencing, and targeted next-generation sequencing (tNGS) have revolutionized TBM diagnosis by providing rapid and accurate pathogen detection. GeneXpert offers high specificity (100%) but moderate sensitivity (46.5%−71.1%) and delivers results within two hours, making it a valuable tool for rapid decision-making [28]. Nanopore sequencing demonstrates high sensitivity (77.78%) and specificity (100%), with a 10-hour turnaround time, making it effective in low-bacterial-load cases and for identifying drug resistance mutations [29,30].

tNGS, combined with machine learning (ML), achieves superior sensitivity (97.01%) and specificity (95.65%) in CSF samples, outperforming traditional methods like Xpert Ultra. It also provides a non-invasive diagnostic alternative using plasma samples with 92.45% sensitivity [31]. Beyond detection, tNGS enables comprehensive drug resistance profiling by identifying mutations in key antitubercular drugs like rifampicin and isoniazid [32]. Its turnaround time of three days further enhances its utility in guiding treatment decisions [32].

#### Imaging and Radiological Techniques:

Magnetic resonance imaging (MRI) remains the imaging modality of choice for TBM. Key findings include basal meningeal enhancement, hydrocephalus, and infarctions. Advanced techniques like diffusion tensor imaging (DTI) detect subtle microstructural changes in white matter tracts, offering insights into disease severity and prognosis [33]. Reduced fractional anisotropy (FA) values in tracts like the corpus callosum correlate with TBM severity and outcomes, highlighting the potential of DTI for earlier diagnosis [34].

#### Emerging Technologies:

Innovative diagnostic tools, such as loop-mediated isothermal amplification (TB-LAMP) and nanopore-targeted sequencing (NTS), are promising in resource-limited settings. TB-LAMP, with its 100% sensitivity and 87.66% specificity, provides results within an hour and requires minimal infrastructure, making it particularly suitable for resource limited settings [35,36]. NTS achieves 60.0% sensitivity and 95.5% specificity for intracranial TB diagnosis, with a 10-hour turnaround time. Its versatility across imaging types and effectiveness in low-bacterial-load cases make it a valuable tool for early and targeted treatment [37].

### Diagnostic Challenges

Despite advancements, diagnosing TBM remains complex, particularly in early stages and in HIV co-infected patients where typical findings may be absent. The Lancet Consensus Scoring (LCS) system offers a practical and cost-effective approach, categorizing TBM cases into definite, probable, and possible based on clinical, CSF, imaging, and other parameters [38]. The system demonstrated a sensitivity of 81.82% and specificity of 100% when compared to MGIT liquid culture, with a diagnostic accuracy of 97.33% [38]. Although its lower sensitivity limits standalone use, the LCS system is a valuable tool when combined with molecular or microbiological tests, particularly in resource-limited settings [38].

### Management of Tuberculous Meningitis

Effective management of tuberculous meningitis (TBM) requires a multifaceted approach combining pharmacological therapies, host-directed treatments, surgical interventions, and non-drug therapies to address complications such as hydrocephalus and drug resistance.

Currently, the baseline regimen typically includes rifampicin, isoniazid, pyrazinamide, and ethambutol for an intensive phase of two months, followed by a continuation phase with rifampicin and isoniazid for 10 months [39]. Adjunctive corticosteroids, such as dexamethasone, are commonly used during the initial phase to reduce inflammation and intracranial pressure, improving outcomes in severe cases [40].

### Advanced Pharmacological Therapies

**Figure 2:**
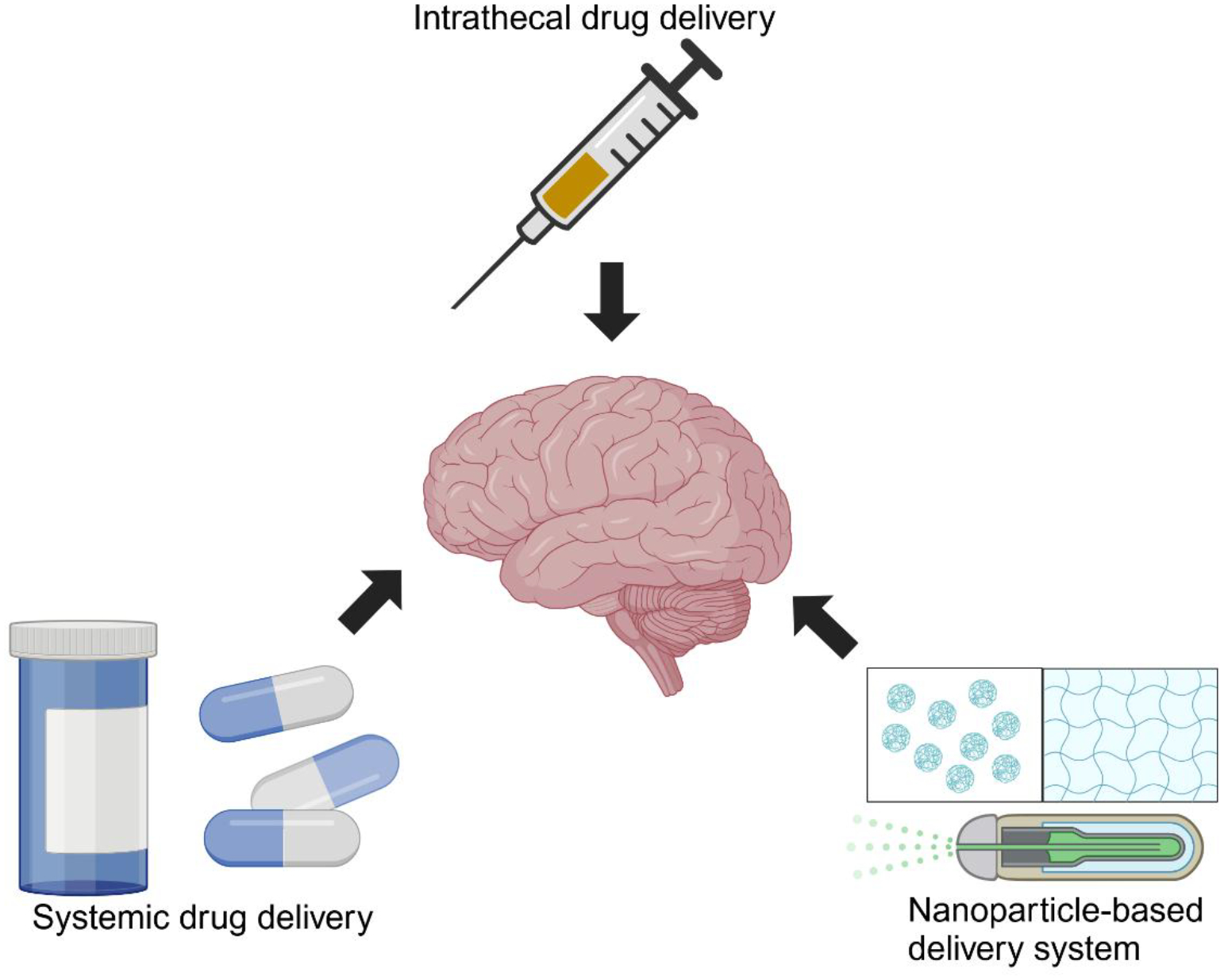
Drug delivery strategies for the treatment of TBM. Systemic drug delivery is the current standard treatment. Intrathecal drug delivery directly delivers medications into the CSF, bypassing the BBB and enhancing CNS penetration. Nanoparticle-based delivery system utilizes hydrogels and osmotic pumps to improve drug bioavailability and targeted release within the brain, reducing systemic toxicity and enhancing treatment efficacy. Created with BioRender.com

Intrathecal therapies, such as isoniazid and steroids, have shown promise in achieving higher drug concentrations in the CSF, enhancing treatment efficacy in refractory TBM cases (Figure 2). For instance, intrathecal administration of isoniazid (100 mg) and prednisolone (20 mg) has rapidly resolved severe meningeal irritation and improved CSF findings [41]. This approach is particularly beneficial when systemic therapies fail to control symptoms [41]. While isoniazid achieves high CSF penetration systemically (80–90%), intrathecal delivery enhances bactericidal effects and minimizes systemic toxicity [41].

A 2024 meta-analysis highlighted the benefits of intrathecal dexamethasone combined with isoniazid (IDI), which showed superior efficacy with a treatment success rate of 91% compared to 70% with standard anti-TB therapy and significantly reduced adverse reactions [42]. IDI therapy improved CSF parameters, including reduced leukocyte counts and protein concentrations, while accelerating recovery from symptoms like fever, headache, and coma [42]. Dose-dependent benefits were observed, with higher doses yielding faster clinical improvements [42].

Nanoparticle-based delivery systems, including hydrogels and osmotic pumps, provide sustained localized drug release, reducing the frequency of dosing and minimizing systemic toxicity [43]. Emerging systems, such as implantable devices for prolonged intrathecal drug release, offer the potential to enhance patient compliance and reduce invasive procedures [43]. These advanced delivery methods optimize therapeutic concentrations and address limitations of traditional anti-TB regimens [43] (Figure 2).

### Host-Directed Therapies

Host-directed therapies (HDTs) targeting immune modulation have emerged as a promising strategy for TBM management. Glutathione (GSH) therapy enhances bacterial clearance by modulating macrophage activity and reducing oxidative stress, with liposomal formulations improving CNS delivery [44]. Combining GSH with N-acetylcysteine (NAC) further reduces oxidative stress and enhances therapeutic outcomes [44]. Vaccine strategies targeting GSH production represent a novel approach for combating multidrug-resistant TBM [44].

Dysregulated immune responses contribute significantly to TBM morbidity, presenting opportunities for targeted interventions. Modulating pathways such as cytokine responses, inflammasome activation, the glutamate-GABA cycle, and the tryptophan pathway shows promise in reducing CNS inflammation and maintaining immune homeostasis [45]. Advanced diagnostics, including CSF immune profiling and single-cell RNA sequencing, aid in identifying inflammatory patterns and enabling precision therapies [45].

Emerging immunomodulatory agents, such as those targeting early cytokine responses and specialized pro-resolving mediators, have shown potential in mitigating inflammation. Additionally, studies highlight the importance of genetic immune factors and their role in host-pathogen interactions, underscoring the need for further clinical validation [45].

### Management of Drug-Resistant TBM

The treatment of drug-resistant TBM necessitates the use of agents with superior CNS penetration, such as linezolid and pretomanid. Pretomanid has demonstrated superior CNS penetration and bactericidal activity, making it a promising candidate for MDR-TBM regimens [44]. When combined with drugs like moxifloxacin and sutezolid, pretomanid has shown potential in reducing bacterial loads in the brain [44].

Recent studies highlight pyrazinamide’s excellent brain penetration and its role in reducing intracerebral inflammation when included in multidrug regimens [46]. However, its use requires careful monitoring due to potential toxicity at elevated CSF concentrations [46]. Advanced imaging techniques, such as PET, are increasingly being used to visualize drug biodistribution in real time, optimizing CNS-specific dosing regimens and improving treatment outcomes [46].

### Linezolid in TBM Management

Linezolid, a bacteriostatic antibiotic from the oxazolidinone class, has shown potential in managing multidrug-resistant TBM (MDR-TBM) [47]. While initial studies indicated benefits such as improved therapeutic concentrations in the CSF, recent research raises concerns about its efficacy and safety. Cross-species studies in rabbits and mice found no improvement in bactericidal activity when linezolid was added to first-line rifampin-containing regimens, with some cases showing higher bacterial burdens [48]. Additionally, CNS penetration of linezolid was lower than previously estimated and declined further after two weeks of treatment, limiting its sustained effectiveness [48].

Clinical trials, such as LASER-TBM, confirmed these findings, showing no significant mortality benefit from linezolid and an increased incidence of adverse events. These outcomes suggest potential drug-drug interactions and emphasize the need for careful consideration of linezolid in treatment protocols. While it may still hold promise for MDR-TBM, its role in first-line regimens remains controversial, necessitating further research to optimize dosing and combinations [47, 49].

### Surgical Interventions

Surgical procedures play a critical role in managing complications like hydrocephalus. Ventriculoperitoneal (VP) shunting and endoscopic third ventriculostomy (ETV) are commonly employed to relieve intracranial pressure [50]. While both methods are effective, ETV avoids some of the long-term complications associated with VP shunting, such as infections and obstructions. However, ETV requires significant technical expertise and specialized equipment [50].

### Non-Drug Therapies

Non-drug therapies, particularly physiotherapy and rehabilitation, are essential components of TBM management, especially for patients with neurological deficits [51]. Targeted physiotherapy programs, including respiratory exercises, muscle strengthening, balance and coordination training, and nutritional support, have demonstrated significant improvements in functional outcomes and quality of life [51,52]. For instance, intensive physiotherapy protocols have shown marked improvements in muscle strength, mobility, and activities of daily living scores, even in severe TBM cases with complications like hydrocephalus or syndrome of inappropriate antidiuretic hormone secretion [51,52]. Incorporating these therapies into multidisciplinary care enhances recovery and long-term outcomes [51,52].

### Paradoxical Reactions

Paradoxical reactions (PRs) in TBM occur when symptoms worsen, or new lesions develop during appropriate anti-TB therapy. These reactions stem from an exaggerated immune response to M. tuberculosis antigens, leading to increased inflammation and worsening clinical features [53]. Common manifestations include enlarging tuberculomas, hydrocephalus, and strokes [53]. PRs can complicate management by mimicking treatment failure or drug resistance [53].

Management typically involves continuing anti-TB therapy alongside adjunctive corticosteroids to suppress inflammation. For severe cases, immunomodulatory therapies such as infliximab or thalidomide may be employed. Serial imaging and close clinical monitoring are essential for distinguishing PRs from treatment failure or secondary infections, ensuring timely therapeutic adjustments [54].

Thalidomide has shown promise in managing severe PRs by inhibiting TNF-α production, reducing inflammation and tissue damage. Combined with corticosteroids, it has been effective in alleviating granuloma formation and hydrocephalus within three months [55]. Similarly, infliximab, a TNF-α inhibitor, has proven beneficial for refractory PRs unresponsive to corticosteroids, improving motor and speech functions and reducing granulomas [56].

### Prognosis and Prevention

TBM is characterized by high mortality and significant neurological effects, with prognosis influenced by a combination of clinical, biochemical, and imaging factors. Systemic inflammation plays a critical role, with elevated neutrophil-to-lymphocyte ratio (NLR) identified as a cost-effective biomarker for predicting both immediate and long-term functional disabilities [57]. Elevated post-treatment NLR levels are associated with poor neurological outcomes, highlighting its utility in prognostic models to improve accuracy and guide interventions [57].

Advances in imaging technology have significantly enhanced the ability to predict disease progression. Computer-aided models that integrate brain MRI data with clinical assessments achieve high accuracy in detecting TBM-associated lesions, such as hydrocephalus, tuberculomas, and vasculitis, with some models demonstrating a 96% accuracy rate [58]. These tools also effectively track disease severity over time and have proven robust across diverse populations, including those co-infected with HIV [58]. Machine learning approaches, achieving 80% accuracy in detecting changes in symptom severity, offer dynamic monitoring capabilities that facilitate timely clinical interventions [58].

CSF biomarkers further contribute to prognosis. Low glucose levels (<1.90 mmol/L) and elevated adenosine deaminase (ADA) levels (>4.80 U/L) are strong predictors of complications such as contralateral isolated lateral ventricle (CILV) following ventriculoperitoneal shunting, emphasizing their value in preoperative and postoperative monitoring [59]. Additionally, older age and advanced disease severity, such as BMRC grade III, are linked to significantly worse outcomes [60]. Drug resistance, particularly to rifampicin, remains a challenge, with resistance identified in 50% of cases [60]. However, effective alternative regimens have mitigated its impact on outcomes in specific settings [60].

Immune markers have emerged as vital prognostic tools. Reduced CD4+ T-cell counts and elevated interleukin-8 (IL-8) levels are independently associated with adverse outcomes in TBM [61]. Improvements in these markers during therapy often correlate with better clinical outcomes, underscoring their value as both prognostic indicators and therapeutic targets [61]. Elevated IL-8 levels also reflect underlying inflammation, further emphasizing the need for targeted therapeutic strategies [61].

Prevention strategies aim to reduce disease incidence, minimize complications, and improve overall outcomes. Early diagnosis remains central, with advanced diagnostic tools combining imaging, inflammatory markers, and artificial intelligence to enhance diagnostic accuracy and facilitate early risk stratification [62]. Neuroimaging markers, including meningeal enhancement and hydrocephalus, have proven valuable in predicting complications such as acute ischemic stroke (AIS), which affects approximately 20% of TBM patients within the first 30 days of admission [62]. Predictive models incorporating these markers provide enhanced sensitivity and critical insights for high-risk patient management [62].

Addressing modifiable risk factors such as diabetes, hypertension, and smoking is essential to prevention [62]. Effective management of these comorbidities significantly reduces the risk of secondary complications [62]. Additionally, advanced age and reduced consciousness at admission are independent predictors of mortality, further underscoring the importance of early recognition and intervention [63].

Recent studies have highlighted the diagnostic and prognostic utility of CSF immunoglobulins (Igs), including IgG, IgM, and IgA, in TBM management. Elevated levels of these immunoglobulins are strongly associated with worse outcomes and more severe cranial MRI findings, such as granulomas and meningeal enhancement [64]. Notably, their significant decline after 24 weeks of treatment highlights their potential for monitoring therapeutic efficacy [64]. When combined, CSF Ig levels offer robust diagnostic accuracy and aid in distinguishing TBM from other central nervous system infections, such as cryptococcal meningitis [64]. These findings suggest that CSF Igs can serve as routine monitoring tools, guiding clinicians in tailoring interventions and improving patient outcomes [64].

### Conclusion and Outstanding Questions

TBM remains a devastating form of extrapulmonary tuberculosis with significant morbidity and mortality, especially in low-resource settings. Despite advances in diagnostics, management strategies, and understanding of its pathogenesis, the disease continues to pose challenges due to delayed diagnosis, treatment resistance, and severe neurological effects. The integration of next-generation diagnostics, HDTs, and innovative drug delivery systems offers promising avenues for improving outcomes. However, the complexity of the disease, compounded by socioeconomic disparities and the emergence of drug resistance, underscores the need for multidisciplinary efforts. Strengthening healthcare systems, enhancing diagnostic accessibility, and advancing research on TBM-specific therapeutic approaches are critical to reducing the global burden of this disease.

Several critical questions remain unanswered in the fight against TBM. A major gap lies in optimizing antitubercular therapies to achieve better brain penetration and faster bacterial clearance, given that current regimens are adapted from pulmonary tuberculosis treatments and may not address the unique challenges of TBM. Additionally, the growing issue of drug resistance raises the need to explore novel agents such as bedaquiline and pretomanid, particularly their efficacy in drug-resistant TBM and their ability to achieve therapeutic concentrations in the central nervous system [44,45]. HDTs also present an important area for investigation. While corticosteroids remain the standard anti-inflammatory treatment, their efficacy in specific populations, such as people living with HIV, is uncertain. Biological agents, including TNF inhibitors and IL-1 receptor antagonists, may offer targeted approaches to mitigate inflammation and improve outcomes [44]. Furthermore, adjunctive therapies aimed at modulating the immune response through specialized mediators like eicosanoids and inflammasomes hold promise for reducing systemic and local inflammation [45].

Diagnostics and prognostic tools for TBM remain underdeveloped, particularly in differentiating TBM from bacterial meningitis. While high-throughput methods like single-cell RNA sequencing and novel biochemical markers have shown promise in enhancing diagnostic precision, their clinical applicability in resource-limited settings remains a challenge [45]. Addressing these disparities through scalable, cost-effective technologies is essential for improving early diagnosis and management. Lastly, clinical trials for TBM often suffer from small sample sizes and poor generalizability. Coordinated global trials with robust designs are essential to evaluate novel therapeutic regimens and interventions [44]. Strengthening research infrastructure in low- and middle-income countries, along with validating new diagnostic and therapeutic approaches in diverse cohorts, is critical to tackling this neglected yet devastating disease [45].

## Keypoints:

- Tuberculous meningitis (TBM) accounts for only 1% of tuberculosis cases but disproportionately contributes to TB-related deaths, with the highest mortality observed in sub-Saharan Africa due to healthcare disparities.
- TBM primarily affects immunocompromised individuals, particularly those with HIV, and populations in low-resource settings, where malnutrition and delayed diagnosis exacerbate outcomes.
- The disease involves Mycobacterium tuberculosis breaching the blood-brain barrier, forming granulomas, and inducing neuroinflammation through mechanisms such as oxidative stress and cytokine dysregulation.
- The nonspecific clinical presentation and paucibacillary nature of TBM make diagnosis difficult, but advances like next-generation sequencing, molecular diagnostics, and advanced imaging have improved accuracy.
- Standard treatment involves multidrug regimens (rifampicin, isoniazid, pyrazinamide, ethambutol) with adjunctive corticosteroids. Intrathecal therapies and nanoparticle-based drug delivery systems are emerging as promising approaches.
- Targeting immune modulation, oxidative stress, and specific pathways such as the glutamate-GABA cycle offers potential for reducing neuroinflammation and improving outcomes in TBM.
- Multidrug-resistant TBM remains a significant challenge, necessitating alternative treatments like linezolid and pretomanid, though these require further research to optimize efficacy and safety.
- Early diagnosis, addressing modifiable risk factors like malnutrition and diabetes, and improving access to advanced diagnostics and therapies are crucial for reducing the global impact of TBM.
